# The Potential Role of FREM1 and Its Isoform TILRR in HIV-1 Acquisition through Mediating Inflammation

**DOI:** 10.3390/ijms22157825

**Published:** 2021-07-22

**Authors:** Mohammad Abul Kashem, Hongzhao Li, Lewis Ruxi Liu, Binhua Liang, Robert Were Omange, Francis A. Plummer, Ma Luo

**Affiliations:** 1 Department of Medical Microbiology and Infectious Diseases, University of Manitoba, Winnipeg, MB R3E 0J9, Canada; kashemm@myumanitoba.ca (M.A.K.); hongzhaoli.ca@gmail.com (H.L.); umliu29@myumanitoba.ca (L.R.L.); plummerf@cc.umanitoba.ca (F.A.P.); 2 JC Wilt Infectious Diseases Research Centre, Winnipeg, MB R3E 0J9, Canada; umliangb@gmail.com; 3 National Microbiology Laboratory, Public Health Agency of Canada, Winnipeg, MB R3E 0J9, Canada; 4 Department of Biochemistry & Medical Genetics, University of Manitoba, Winnipeg, MB R3E 0J9, Canada; 5 Vaccine and Gene Therapy Institute, Oregon Health and Science University, Portland, OR 97006, USA; romangew@gmail.com

**Keywords:** FREM1, TILRR, inflammation, HIV-1 acquisition

## Abstract

FREM1 (Fras-related extracellular matrix 1) and its splice variant TILRR (Toll-like interleukin-1 receptor regulator) have been identified as integral components of innate immune systems. The potential involvement of FREM1 in HIV-1 (human immunodeficiency virus 1) acquisition was suggested by a genome-wide SNP (single nucleotide polymorphism) analysis of HIV-1 resistant and susceptible sex workers enrolled in the Pumwani sex worker cohort (PSWC) in Nairobi, Kenya. The studies showed that the minor allele of a FREM1 SNP rs1552896 is highly enriched in the HIV-1 resistant female sex workers. Subsequent studies showed that FREM1 mRNA is highly expressed in tissues relevant to mucosal HIV-1 infection, including cervical epithelial tissues, and TILRR is a major modulator of many genes in the NF-κB signal transduction pathway. In this article, we review the role of FREM1 and TILRR in modulating inflammatory responses and inflammation, and how their influence on inflammatory responses of cervicovaginal tissue could enhance the risk of vaginal HIV-1 acquisition.

## 1. Introduction

FREM1 (Fras-related extracellular matrix 1) plays a critical role in epithelial–mesenchymal interactions [1]. It forms a ternary complex with other integral membrane proteins including FRAS1 (Fraser syndrome 1) and FREM2 [2]. FREM1 functions as an integral component in embryonic development [3], whereas FREM1 variant TILRR (Toll-like interleukin-1 receptor regulator) acts as a modulator of innate immune responses and inflammation [4]. Mutations of FREM1 are associated with multiple phenotypic abnormalities [5]. Because FREM1 is an extracellular matrix (ECM) protein, it may interact with other neighboring ECM partners and promote cell adhesion, migration, proliferation, and differentiation. Studies also showed that TILRR, a splice variant of FREM1, is an IL-1R1 (interleukin 1 receptor, type 1) co-receptor and a highly conserved membrane-associated glycosylated protein [4]. The interactions of TILRR with IL-1-IL-1R1 potentiate the activation of NF-κB transcription factor and inflammatory responses [4,6]. The potential involvement of FREM1 and its variant TILRR in vaginal HIV-1 acquisition was brought about through a genome-wide SNP (single nucleotide polymorphism) analysis. We conducted the study to identify genetic factors associated with the natural resistance to HIV-1 infection observed in a sub-group of female sex workers enrolled in the Pumwani sex worker cohort [7,8,9,10]. The Pumwani sex worker cohort, an open prospective cohort study of sexually transmitted infections (STIs), was established in Nairobi, Kenya in 1985 and the patients enrolled (n > 5000, 1985–2012) in the cohort have been followed biannually since the cohort’s establishment [11,12,13,14]. A sub-group of women enrolled in the cohort remains HIV-1 uninfected despite repeated high-risk exposure through sex work [14]. The genome-wide SNP analysis identified an SNP rs1552896 in FREM1, and its minor allele is enriched in HIV-resistant women [15]. Our subsequent studies showed that FREM1 mRNA is highly expressed in tissues that are relevant to vaginal HIV-1 infection [15]. Furthermore, the FREM1 variant, TILRR, increased the expression of many genes in the IL-1-NF-κB inflammatory signaling pathway [16]. It has been shown that the IL-1-NF-κB signaling pathway functions as an enhancer of HIV-1 acquisition/susceptibility via recruitment and/or activation of HIV-1 target cells [17]. Genital inflammation accompanied by increased activated CD4+T-cells is associated with increased HIV-1 acquisition risk in South African young women [18,19]. Since HIV-1 acquisition is supported by the inflamed genital mucosa where inflammatory mediators fuel the recruitment and activation of HIV-1 target cells [20], it is possible that FREM1 and its variant, TILRR, may be involved in HIV-1 vaginal acquisition/infection through the regulation of inflammatory responses. It is important to emphasize that activated CD4+ T cells support the productive HIV-1 infection [21,22,23]. An inflammatory environment in the female genital tract (FGT) would have a higher amount of activated CD4+ T cells [17,24,25], thus helping HIV-1 to establish and expand infection. Therefore limiting inflammatory responses could reduce the risk of HIV-1 vaginal acquisition. Thus, studying mediators of inflammatory responses, such as TILRR, may help to identify novel HIV-1 prevention targets. In this article, we will review the literature about the FREM1 and TILRR and their role in inflammation responses and inflammatory diseases, and discuss their potential role in HIV-1 vaginal acquisition.

## 2. Literature Search Strategy

We conducted an exhaustive search for available publications relevant to FREM1 and TILRR in five different databases including PubMed/Medline (OVID), EMBASE, Web of Science, Scopus, and Google Scholar through the University of Manitoba Library (https://libguides.lib.umanitoba.ca/az.php Accessed on 25 May 2021). We included keywords, such as FREM1, TILRR, and FREM1/TILRR in inflammation for the search. The focus of this review was FREM1/TILRR; however, we also included additional publications that were relevant to the topic of interest such as literature on genital inflammation and HIV-1 infection. The literature between 1981 and 2020 was included because FREM1 and its variants were identified in humans in 2001, and HIV-1 was first reported in 1981. In addition to the published literature, we also included some unpublished data from our lab. All eligible literature was screened using guidelines of PRISMA (Preferred Reporting Items for Systemic Reviews and Meta-analysis) [26]. We used PRISMA as a guideline to select published literature in this review. Conference abstracts and data-only files that are part of full publications were excluded. All relevant studies were included in this review (Supplementary Figure S1 and Table S1).

## 3. Fras-Related Extracellular Matrix 1 (FREM1)

### 3.1. Molecular Structure of FREM1 and Its Splice Variants

**Figure 1:**
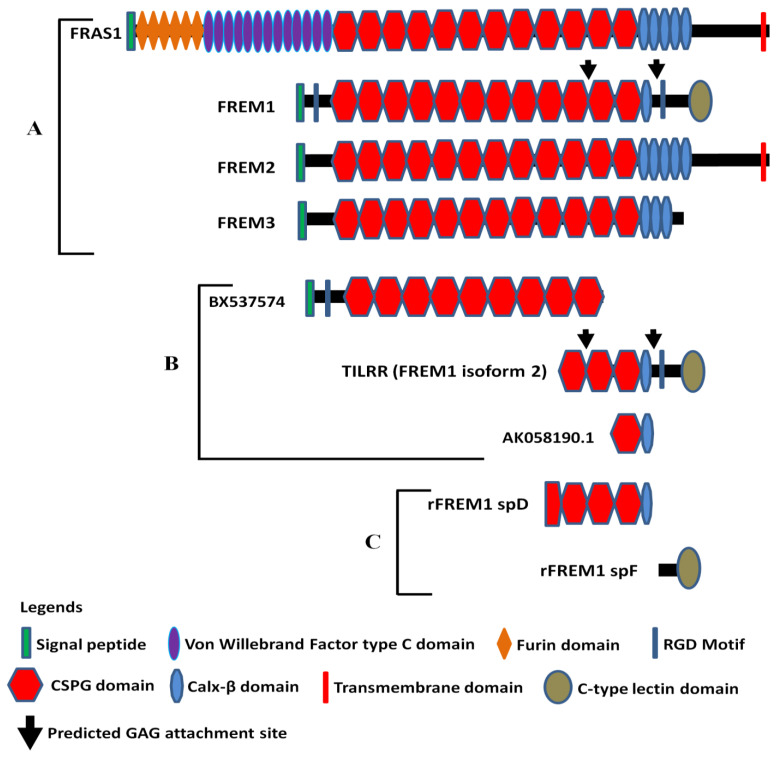
Diagram of FRAS/FREM family proteins, FREM1 splice variants (TILRR and others), and recombinant FREM1 proteins. (**A**) FRAS/FREM family proteins, including FREM1 (FREM1 isoform 1); (**B**) TILRR (FREM1 isoform 2) and other truncated variants of FREM1 identified in PBMCs (peripheral blood mononuclear cells) of women enrolled in the Pumwani sex worker cohort; (**C**) Recombinant FREM1 proteins (rFREM1 spD and rFREM1 spF). RGD, arginine-glycine-aspartic acid; CSPG, chondroitin sulfate proteoglycan; and GAG, glycosaminoglycan. This figure was adapted with permission from Kashem et al. [16], Short et al. [1], and Yuan et al. [29].

Full-length human FREM1 contains multiple functional domains that interact with extracellular matrix molecules. It has an N-terminal signal sequence, positioned at 1–23 of its amino acid (aa) sequence, and an N-terminal variable region (NV)-containing domain (from aa 24–274) with an Arg-Gly-Asp (RGD) motif (from aa 199–201). Following the NV domain, there are 12 chondroitin sulfate proteoglycan (CSPG) tandem repeats. Each CSPG domain is approximately 120 aa long (from aa 275–1730) [27,28]. At the C-terminal region, it contains a calcium-binding loop of approximately 89 aa long (Calx-β domain, from 1731–1820 aa), and an RGD motif sequence (from 1907–1909 aa). At the end of the C-terminus, it has a unique type C lectin-like (LecC) domain, which is ~131 aa long (from aa 2052–2179) [27]. Additionally, it bears two glycosaminoglycan (GAG) attachment sites located between CSPG10 and CSPG11 domains, and Calx-β and C-terminal RGD motif, which were shown to bind to the IL-1R1 receptor of IL-1-NF-κB signaling pathway (Figure 1A) [16,29].

The amino acid sequence of N-terminal signal peptide is highly variable between human and mouse FREM1 protein and, therefore, termed as N-terminal variable region-containing domain (NVD) [27]. FREM1 RGD motif sequence is a well-studied cell-adhesive motif and functions as an integrin-binding region. It is recognized by a wide group of integrins, such as αv subunits (αvβ1 and αvβ3) and the β1 subunits (α5β1 and α8β1), which transduce signals to the cells [27]. Twelve CSPG repeats, also known as cadherin repeat-like repetitive elements [3,28] initially identified in NG2 (neural/glial antigen 2) core protein [30], are the hallmark of Fras/FREM family proteins because they are common to all members of the family [3]. Studies have demonstrated that NG2 core proteins (CSPG motifs) frequently interact with an array of extracellular matrix (ECM) proteins, including collagen type II, V, and VI [31,32,33], platelet-derived growth factor (PDGF-AA, a longer isoform of PDGF) [34], and basic fibroblast growth factor (bFGF2) [34]. Thus, it is suggested that FREM1 binds to ECM proteins of basement membranes (BMs) through its repetitive CSPG domains [35] to conduct its biological functions, including cell adhesion, cell proliferation, and migration. The Calx-β domain of FREM1 is a calcium-binding loop of Na^+^-Ca^+^ exchange β. The Calx-β domain is characterized by a cytoplasm-localized, calcium chelating, and calcium-binding region of transmembrane proteins, such as integrin β4 [36]. Additionally, the unique feature of FREM1 protein is having a C-terminal LecC domain (CTLD) which is associated with carbohydrate residues of ECM. The other members of Fras/FREM family proteins do not have CTLD [1].

According to AceView [37,38], FREM1 has approximately 15 splice variants. Of them, a full-length isoform 1 encoding the 2179 amino acids long full FREM1 protein (NM_144966.5/NP_659403.4), and a shorter isoform 2 encoding a 715 aa long TILRR protein (NM_001177704.1/NP_001171175.1) were described [35]. The FREM1 isoform 2 (TILRR) has a structurally different N-terminal region and only has 3 CSPG domains with a Calx-β domain and a C- terminal LecC domain [16,29]. Similar to isoform 1, it contains an RGD motif and two GAG attachment sites that are associated with cell adhesion and IL-1β signal transduction, respectively (Figure 1B). In addition, RNA-seq analysis of PBMCs (peripheral blood mononuclear cells) further identified two truncated FREM1 isoforms (BX737574 and AK058190.1) in women of the Pumwani sex worker cohort (PSWC), Nairobi, Kenya (Figure 1B). Unlike FREM1 and its splice variants, the structures of other Fras/FREM-family members are quite different in their N- and C-terminal regions (Figure 1A).

### 3.2. FREM1 Expression, Localization, and Interactions

FREM1 protein is expressed in a wide variety of human and animal cells and tissues. It functions as an important extracellular molecule either in developing embryos or in the pathogenesis of multiple abnormalities. FREM1 is primarily secreted by the mesenchymal cells underlying the basement membrane of visceral pleura and in epithelial and mesothelial cells [39,40]. The protein is localized in the dermal layer where it forms a stable complex with other Fras/FREM-related proteins called ternary complexes [2]. Although both dermal and epidermal cells express FREM1 [1,40], its expression is most prominent in dermal cells of the eyelids, head, and limbs of mice [3]. It is also expressed in the diaphragm [39], intestine, lung, kidney, and skin [40], as well as craniofacial structures, such as ears, forehead, midface, nose, teeth, and hair follicles in mice [3,41,42].

A previous study from our group showed that mRNA of FREM1 is expressed in various human tissues, including cervix, colon, esophagus, heart, kidney, lung, ovary, placenta, prostate, skeletal, small intestine, testes, thymus, thyroid, and trachea [15]. The expression of FREM1 was observed at a high level in cervical tissues, the small intestine, and kidneys. Immunohistochemistry staining of human ectocervical biopsy specimens from two different groups of women (HIV-1 negative new enrollees and HIV-1 resistant) of the Pumwani sex worker cohort demonstrated that FREM1 protein is expressed in the epithelial and sub-mucosal layer underneath the basement membrane (BM), particularly the upper lamina propria [15].

### 3.3. FREM1 and Immune Cell Infiltration

Extracellular matrix (ECM) proteins function as mediators of inflammation and infiltration of immune cells [43,44,45,46]. Because FREM1 is an extracellular matrix protein and widely expressed in the regions of epithelial–mesenchymal interaction [3], its expression in epithelial tissues may promote immune cell infiltration. Using immunohistochemistry (IHC) and immunofluorescence (IF) assays, a study demonstrated that elevated expression of FREM1 in normal breast/mammary epithelial tissues is positively correlated with the infiltration of several immune cells, such as CD4+, and CD8+T -cells, and M1 (inflammatory) macrophages [47]. In addition, analysis of breast cancer (BC) epithelial tissues showed that high FREM1-expressed BC tissues harbor abundant CD4+T memory cells (resting and activated), CD8+T-cells, M1 macrophages, resting dendritic cells, gamma-delta T cells (γδT), B-cells (naive and memory), plasma cells, and resting mast cells. Contrastingly, low FREM1-expressed BC tissues tended to have a higher proportion of M2 (anti-inflammatory) macrophages, neutrophils, and resting NK (natural killer) cells [47]. It has also been shown that the RGD motif of ECM binds with integrins and promotes migration of effector T cells into the interstitial inflamed tissues [48]. Thus, the binding of FREM1 with integrins may function as a critical regulator in infiltrating immune cells, especially in cervicovaginal tissues. Although the direct role of FREM1 in infiltrating immune cells in cervical tissues is yet to be examined, the expression of FREM1 in the cervix of humans [15] supports the notion that FREM1 may function as an inducer of immune cell infiltration, including HIV-1 target cells, facilitating vaginal HIV-1 infection.

### 3.4. FREM1 Polymorphism and HIV-1 Resistance

Pumwani sex worker cohort (PSWC) was established in the heart of Pumwani slum, Nairobi, Kenya in 1985. It is an open prospective cohort study of the immunobiology and epidemiology of sexually transmitted infections (STIs), and the patients enrolled in the cohort have been followed biannually since the cohort’s establishment [11,12,13,14]. This cohort is not only involved in the research, but also provides services related to STIs and HIV-1 prevention and care, such as consultation, provision of free condoms, and treatment of other infections. Between 1985 and 2012, more than 5000 sex workers have been enrolled in the cohort. A sub-group of women in PSWC remained HIV-1 uninfected despite the frequent exposure to high-risk HIV-1 infected sexual partners [14]. These women demonstrated a significantly high-level expression of various potentially HIV-1 inhibitory molecules, including RANTES (regulated on activation, normal T-cell expressed and secreted) [49], serpins, elafin, and many other factors [49,50,51] at their genital mucosa. Multiple immunological [52,53,54,55], genetic [7,8,9,10,56], and proteomics [50,51] factors are associated with this phenotype.

Our group has conducted a genome-wide analysis of SNP (single nucleotide polymorphism) among women of PSWC to identify the genetic polymorphism associated with the HIV-1 resistant phenotype [15]. The study showed that the minor allele of FREM1 SNP rs1552896 is enriched in HIV-1 resistant women and the major allele of the SNP rs1552896 is associated with HIV-1 susceptible women [15]. Furthermore, the frequency of the minor allele of rs1552896 was also found to be higher in HIV-1 uninfected women in comparison to HIV-1 infected individuals in a mother–child HIV-1 transmission (MCHT) cohort [15]. Thus, the minor allele of FREM1 SNP rs1552896 is associated with the HIV-1 resistant phenotype.

**Figure 2:**
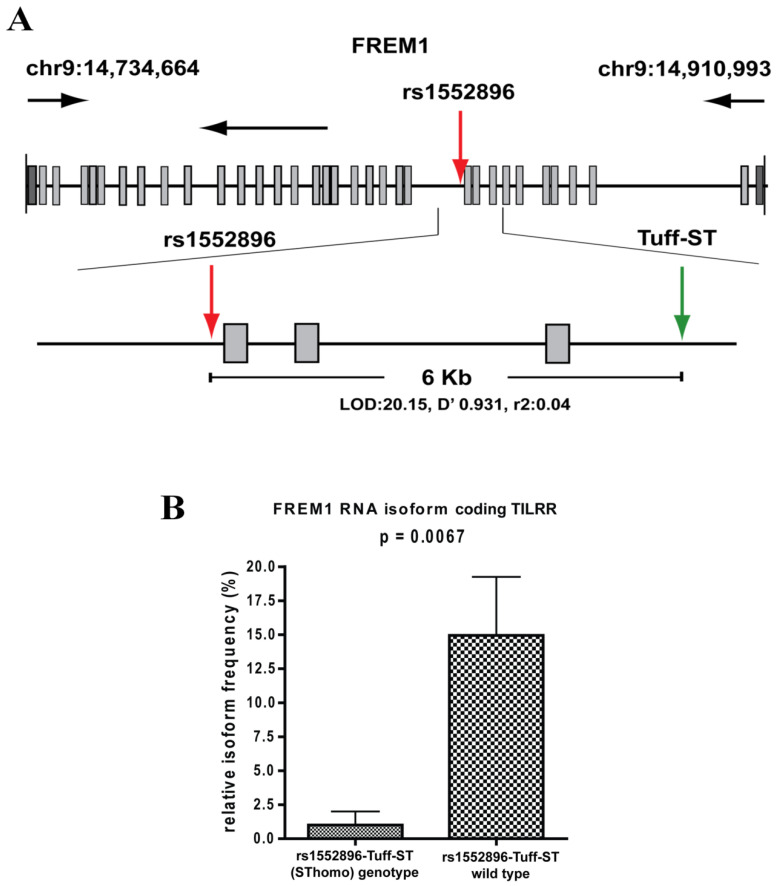
Full gene sequencing of FREM1 and RNA-Seq analysis of PBMCs. (**A**) Full gene sequencing of FREM1 gene of 70 women in the Pumwani sex worker cohort identified a novel microsatellite Tuff-ST that is in linkage disequilibrium with the SNP rs1552896 (LOD 20.15, D′ 0.931, r^2^: 0.04). Further Sanger sequencing and genotype of 1090 women for this microsatellite showed that rs1552896-Tuff-ST (SThomo) genotype is significantly associated with resistant women (*p* = 0.0002). (**B**) RNA-Seq analysis of PBMCs of Pumwani sex workers with different rs1552896-Tuff-ST genotypes. Women with the protective rs1552896-Tuff-ST (SThomo) genotype do not express or express a very low amount of FREM1 RNA isoform encoding TILRR in comparison with women with the rs1552896-Tuff-ST wild type (*p* = 0.0067) (Luo et al., unpublished data).

The SNP, rs1552896, is located at the intron 11 (57 bp) close to the 3′ end of the exon 11 of the FREM1 gene. The association of its minor allele with resistance to HIV-1 infection may be due to its influence in alternative splicing of FREM1, and/or it may be a marker for polymorphisms in the coding region that affects the structure and function of FREM1. To clarify this, we conducted full gene sequencing and a pilot RNA-Seq analysis in the women of the Pumwani sex worker cohort. Further genetic analysis identified a novel microsatellite (Tuff-ST) in LD (linkage disequilibrium) with rs1552896, and the allele SThomo of Tuff-ST microsatellite is strongly associated with HIV-1 resistant women and has a strong likelihood of modifying FREM1 transcript alternative splicing (Figure 2A) (Luo et al., unpublished data). Pilot RNA-Seq analysis supported this possibility and showed that the FREM1 isoform coding for TILRR was detected in most women with rs1552896-Tuff-ST wild type. However, this isoform is either absent or expressed at a very low level in women with the protective rs1552896-Tuff-ST (SThomo) genotype (*p* = 0.0067) (Figure 2B) (Luo et al., unpublished data). Based on these supporting data, we hypothesize that FREM1 plays an important role in the vaginal transmission of HIV-1 through the regulation of genes involved in immune activation, inflammatory responses, mucosal integrity, and cell migration. The association of the rs1552896-Tuff-ST (SThomo) genotype with resistance to HIV-1 infection may be partly due to an impaired IL-1R1/TLR signal transduction resulting from the abolition/reduced TILRR isoform expression.

### 3.5. Monoclonal Antibodies to Study FREM1 and Its Splice Variant TILRR

Antibodies are important tools to study the function of proteins and have been used as therapeutics for many diseases [57]. To study the function of FREM1 and its role in HIV-1 infection, our group developed anti-FREM1 antibodies [29]. To develop anti-FREM1 monoclonal antibodies, we expressed two recombinant proteins of FREM1, rspD (376 amino acids, 56.8 kDa) and rspF (255 amino acids, 29.5 kDa). The two recombinant proteins overlap the C-terminal region of full-length FREM1 isoform 1 and the majority portion of FREM1 isoform 2 (TILRR). Structurally, rspD protein contains a small portion of the CSPG9 domain as well as CSPG10, CSPG11, CSPG12, and Calx-β domains of FREM1, whereas rspF only contains C-type Lectin domain (CTLD) (Figure 1C). Our group developed 17 anti-FREM1 mouse monoclonal antibodies (mAbs) and mapped their epitopes [29]. These anti-FREM1 mAbs target major and minor epitopes of multiple domains of FREM1 and its splice variants. These in-house developed anti-FREM1 mAbs and recombinant FREM1 proteins are tools to study the role of FREM1 and its variants in inflammation responses and vaginal HIV-1/SIV (simian immunodeficiency virus) acquisition. We are using this panel of mAbs to study the expression and function of FREM1 and TILRR.

## 4. Toll-like Interleukin -1 Receptor Regulator (TILRR)

### 4.1. Characteristics of TILRR

TILRR is a shorter FREM1 transcript consisting of 715 aa residues [4,58]. It associates with IL-1/IL-1R1 signaling receptor, binds with the host cell membrane by its structural C-terminal LecC domain, and increases the cell surface receptors’ expression and ligand binding activities [4]. As a truncated variant of FREM1, its transcriptional start site is located within an intronic segment between exons 24 and 25 of the FREM1 gene called 5′ TILRR coding sequence (CDS) [59]. The 5′ TILRR CDS was found in human and mouse FREM1 and was, therefore, used to identify ortholog of TILRR in other organisms for their evolutionary history and development. Analysis of 5′ CDS in tetrapods (amphibians), teleosts (fish), and invertebrates (*Drosophila melanogaster, Caenorhabditis elegans*, and *Ciona intestinalis*) showed that only tetrapod organisms possess 5′ TILRR CDS within the FREM1 ortholog. Although teleost organisms possess at least one FREM1 ortholog, no 5′ TILRR CDS was recognized in these organisms [59].

In invertebrates, in contrast, neither FREM1 ortholog nor 5′ TILRR CDS were found. Thus, FREM1 appears following the evolution of vertebrates; however, TILRR was only identifiable after the divergence of teleosts. These suggest that FREM1/TILRR may evolve from the common ancestor of tetrapod and teleost organisms and have subsequently become extinct in FREM1 paralogs prior to the modern teleosts’ evolution [59]. In addition, FREM1/TILRR may emerge due to the divergence of a common ancestor in the tetrapod lineage [59].

### 4.2. TILRR Expression

TILRR is expressed in a wide variety of human and animal cells. The mRNA expression analysis of PBMCs, and several epithelial, endothelial, and other immune cell lines demonstrated that TILRR mRNA was expressed in all cells, but the level of expression is variable [4]. In humans, TILRR mRNA is relatively highly expressed in several cell lines, including human microvascular endothelial cell (HMEC-1), human embryonic kidney cells (HEK293), leukemic cells (K562, ML-1, HL-60, and MOLT-4), and macrophage cell line (U937); however, lower expression of TILRR mRNA was reported in human cervical epithelial cell (HeLa), monocytes (THP-1) and PBMCs. In animals, on the other hand, high levels of TILRR mRNA were observed in murine mammary epithelial cells (C127), fibroblast (3T3), and monocyte/macrophage cells (J774.2), and relatively low expression was reported in a murine macrophage cell line (RAW 264.7). Zhang et al. [4] further demonstrated that TILRR is a 70 kDa protein and its protein expression level is consistent with the level of mRNA expression.

Smith et al. [60] showed that TILRR mRNA is expressed in murine monocyte-derived macrophages (RAW 264.7). A high level of TILRR mRNA expression was also found in PBMCs of myocardial infarction-diagnosed patients [60]. Additionally, immunohistochemistry staining of the carotid artery revealed a significantly elevated level of TILRR expression in the atherosclerotic plaque [60]. A recent study further demonstrated that TILRR mRNA is highly expressed in normal human breast/mammary epithelial cells [58]. Taken together, TILRR mRNA is expressed in multiple immune and epithelial cells in humans and animals, and its expression implicates the importance of TILRR in innate immune responses and inflammatory diseases.

### 4.3. TILRR in Innate Signal Transduction and Inflammation

**Figure 3:**
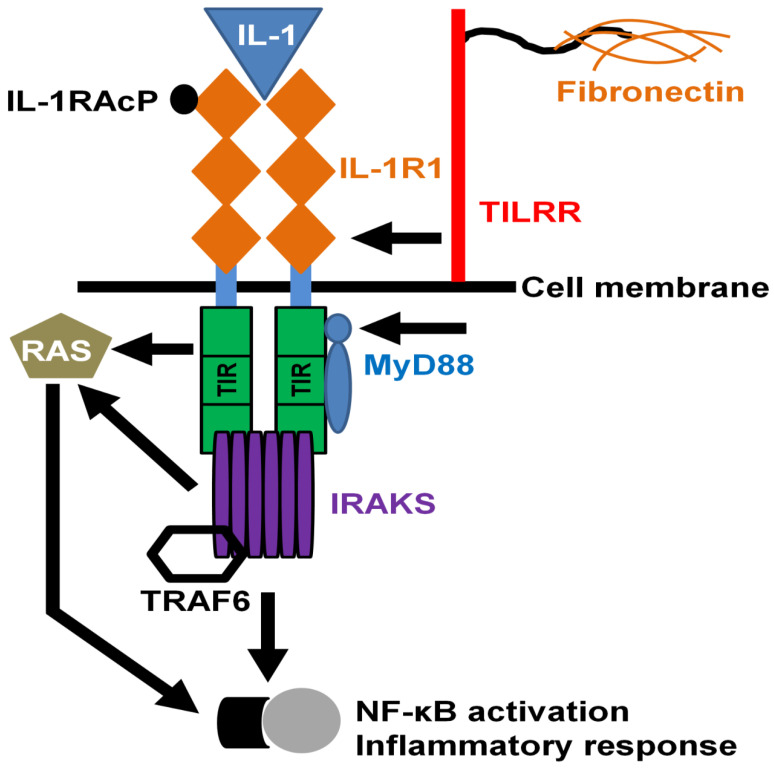
TILRR potentiates MYD88 recruitment to IL-1R1 to induce NF-κB signaling and inflammatory responses. TILRR binds with IL-1R1 as a co-receptor and potentiates the recruitment of MYD88 adapter to the cytoplasmic TIR domain. The association of adapter molecule with TIR domain induces the signal amplification and directs the Ras GTPase-controlled enhancement of NF-κB induction and inflammatory genes. This figure was adapted with permission from Zhang et al. [4].

Toll-like/IL-1 receptors regulate the innate immune and inflammatory responses through association with the IL-1R1 receptor complex followed by engagement of MYD88 (myeloid differentiation primary response 88) adaptor protein to the cytoplasmic TIR (toll/IL-1 receptor) domain, and transcriptional modification of NF-κB transcription factor [61,62,63]. As a co-receptor of IL-1R1, TILRR interacts with the IL-1R1-TIR complex and augments the signal transduction by enhancing ligand-binding activities and the expression of IL-1R1 receptor [4,64]. The association of TILRR with IL-1R1 not only potentiates the receptor expression, but also enhances the recruitment of cytoplasmic MYD88 adapter on the TIR domain, resulting in the expression of pro-inflammatory genes after NF-κB signaling pathway activation (Figure 3) [4,6]. TILRR mediates the activation of inflammatory genes in a wide variety of cells including macrophages, epithelial cells, and fibroblasts via TIR regulatory components [4].

Recently, we have shown that TILRR overexpression increased the expression of numerous NF-κB signal transduction and inflammation-responsive genes [16]. Smith et al. [60] demonstrated that inflammatory conditions, such as lung fibrosis and myocardial infarction, trigger an elevated TILRR expression in blood monocytes/macrophages. It has also been shown that blocking with an anti-TILRR antibody or genetic deletion of TILRR has significantly reduced the monocyte/macrophage population in the inflamed areas [60]. Microarray analysis of blood samples further revealed that knockout of TILRR in mice greatly reduced inflammatory gene expression [60]. Gabhann [65] elucidated that the binding of IL-1 with its cognate receptor IL-1R1 forms the IL-1-IL-1R1 complex, which initiates a medium level of NF-κB activation. In contrast, the association of co-receptor TILRR with IL-1-IL-1R1 complexes, such as IL-1-IL-1R1-TILRR, instigates a higher level of NF-κB activation, resulting in increased pro-inflammatory responses during pathological conditions. Thus, TILRR mediates inflammation and targeting TILRR can reduce its function, which may significantly reduce inflammatory responses, immune cell migration, and infections or disease associated with inflammation. Reducing TILRR level may also lower the risk of HIV-1 acquisition.

### 4.4. TILRR and Production of Inflammatory Mediators

Because TILRR is involved in IL-1-IL-1R1 signal transduction events [4], it may potentiate the production of soluble pro-inflammatory cytokines/chemokines through modulation of mRNA expression of genes associated with the NF-κB signaling pathway. Studies have shown that activation of cytoplasmic sequestered NF-κB transcription factor together with nuclear translocation of NF-κB is the key step to initiate mRNA and protein expression of genes involved in inflammatory responses [66,67]. Rhodes et al. [64] demonstrated that binding of TILRR with IL-1R1 potentiates the release of NF-κB from its cytoplasmic inhibitory molecules, resulting in NF-κB nuclear translocation, activation, and inflammatory cascade reactions. Using TILRR knockout mice, Smith et al. [60] demonstrated that mRNA expression of inflammatory genes encoding MCP-1 (monocyte chemoattractant protein 1)/CCL2, IL-6 (interleukin 6) and TNFα (tumor necrosis factor alpha) was significantly reduced in bone marrow-derived macrophages and splenic samples compared to that of wild type control. A recent study also showed that overexpression of TILRR in BT474, a human breast tumor cell line, significantly potentiates the expression of immune and inflammation-associated genes, including IL-6, IL-8/CXCL8, IP-10 (interferon gamma-induced protein 10)/CXCL10, MCP-1/CCL2, and RANTES/CCL5 [58]. Using human breast tumor tissues, Xu et al. [58] further demonstrated that elevated expression of TILRR is positively correlated with mRNA expression of IP-10/CXCL10. However, these studies did not measure the protein level expression of these genes.

Although we know little about the effect of TILRR on the pro-inflammatory cytokine/chemokine production, the existing data suggested that TILRR may play a crucial role in the production of soluble mediators [4,6,64]. To test this possibility, we overexpressed TILRR in two human epithelial cell lines (HeLa and VK2/E6E7) [16]. The data from our study demonstrated that overexpression of TILRR significantly increased the number of soluble pro-inflammatory cytokines/chemokines (IL-6, IL-8/CXCL8, IP-10/CXCL10, and RANTES/CCL5) in a time-dependent manner in culture supernatants of both cell lines. We further reported that MCP-1/CCL2 and MIP-1β (macrophage inflammatory protein 1 beta)/CCL4 were increased in a cell type-specific manner where MCP-1/CCL2 and MIP-1β/CCL4 were significantly elevated in HeLa and VK2/E6E7 cell lines, respectively. Thus, TILRR modulates pro-inflammatory cytokine/chemokine production by immune cells and cervicovaginal epithelial cell lines, and potentially influences the genital inflammatory environment, and enhances susceptibility to HIV-1 acquisition.

### 4.5. TILRR and Migration of HIV-1 Target Cells

Activation of IL-1-IL-1R1-TILRR-NF-κB signaling complex potentiates the production of inflammatory cytokines/chemokines in immune and non-immune cells including epithelial cell lines [16,60]. However, it is not known whether the TILRR-modulated production of inflammatory mediators promotes the migration of immune cells, including HIV-1 target cells. Production of cytokines/chemokines by epithelial cells initiates the cognate receptor-mediated signal that attracts immune cells into the tissues. Monocyte chemotactic protein (MCP)-1 or CCL2 produced from epithelial cells promotes infiltration of various immune cells (monocytes/macrophages, T-lymphocytes, and NK-cells) through CCR2 receptor expression on target cells [68]. Thus, it is possible that the TILRR-induced production of inflammatory mediators by genital epithelial cells would promote the migration of immune cells. Although currently there is no report on the role of TILRR in promoting immune cell migration in cervicovaginal tissues, a study showed that TILRR influenced the infiltration of monocyte under inflammatory conditions [60]. Recently, another study showed that higher expression of TILRR in breast/mammary epithelial tissues is significantly positively correlated with immune cell infiltration, mostly CD4+ and CD8+ T cells, and to a lesser extent of B cells, macrophages, dendritic cells, and neutrophils [58].

Because TILRR augments the NF-κB signaling pathway via IL-1-IL-1R1 receptor complex [4,6] and potentiates the production of inflammatory mediators from cervicovaginal epithelial cell lines [16], we hypothesized that TILRR-modulated production of cytokines/chemokines may promote migration of immune cells into cervicovaginal tissues. To address this notion, we conducted migration experiments using monocytes (THP-1) and T-lymphocytes (MOLT-4), the model HIV-1 target cells, and TILRR-overexpressed HeLa cell culture supernatants. We examined the migration behavior of cells in Transwell assay and microfluidic device assay. Our data showed that monocytes (THP-1) and T-lymphocytes (MOLT-4) migrated significantly towards the TILRR-overexpressed cell culture supernatants and migrated longer distances [69]. Therefore, the data showed that TILRR-modulated production of soluble mediators can promote migration of immune cells, and have a potential role in inflammation-induced vaginal HIV-1 acquisition by attracting immune cells to the site of inflammation.

### 4.6. Potential Role of FREM1 and Its Isoform TILRR in Vaginal HIV-1 Acquisition

**Figure 4:**
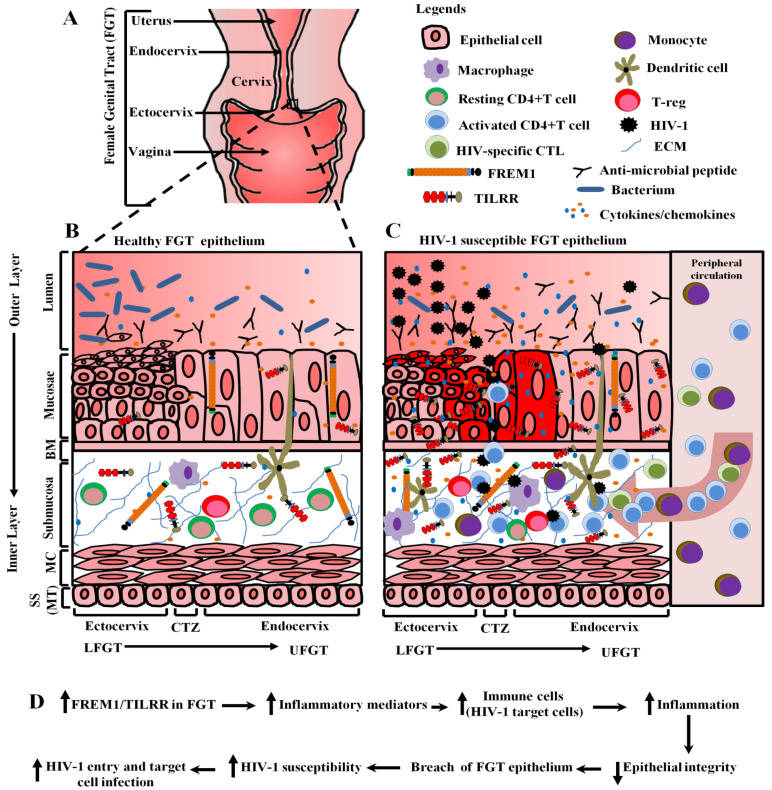
Proposed model on the potential role of FREM1/TILRR in vaginal HIV-1 acquisition. (**A**) A schematic view of the female genital tract (FGT). (**B**) A close-up view of different layers of healthy cervical epithelial tissue. In figure B, the expression of FREM1/TILRR in healthy cervical tissue maintains the epithelial integrity, which prevents the invasion of pathogens including HIV-1 into the genital tissue. (**C**) A close-up view of different layers of HIV-1 susceptible cervical epithelial tissue. In figure C, the high-level expression of FREM1/TILRR increases the production of pro-inflammatory cytokines/chemokines by epithelial cells [16]. The increased production of pro-inflammatory cytokines/chemokines attracts the infiltration of immune cells from the periphery to the cervical tissues, including HIV-1 target cells [24,69,72]. The cytokines secreted by the infiltrated immune cells may further activate and attract more immune cells and resulted in cycles of inflammation and breakdown of epithelial barrier. Studies showed that genital inflammation increases the risk of HIV-1 acquisition [24,25,70]. The proposed model showed that TILRR promotes the production of cytokines/chemokines by epithelial cells and attracts the infiltration of immune cells, including CD4+T-cells and macrophages [58], and it is a risk factor in HIV-1 acquisition. (**D**) A flow diagram shows the effect of FREM1/TILRR in vaginal HIV-1 acquisition through genital epithelium as illustrated in figure C. An upwards arrow represents increased and a downwards arrow indicates decreased. Legends on the upper right corner indicate the various immune components of the female genital tract. FGT, female genital tract; BM, basement membrane; MC, muscular; SS, serosae; MT, mesothelium; CTZ, cervical transformation zone; LFGT, lower female genital tract; UFGT, upper female genital tract; CTL, cytotoxic t-lymphocyte; T-REG, t-regulatory; FREM1, Fras-related extracellular matrix 1; and TILRR, toll-like interleukin-1 receptor regulator; ECM, extracellular matrix; HIV-1, human immunodeficiency virus type 1.

Inflammation is a driving force of HIV-1 acquisition [24,25,70], and the uncontrolled inflammatory response of genes in the IL-1-NF-κB signaling pathway may enhance HIV-1 susceptibility via recruitment and/or activation of HIV-1 target cells [17]. NF-κB transcription factor is one of the central regulators of many inflammatory genes [66] and inflammation [67,71]. NF-κB activation is potentiated by the association of TILRR with IL-1R1 receptor [4,65], and TILRR-associated NF-κB activation instigates the production of inflammatory cytokines/chemokines, and migration of immune cells [16,58,69]. Since cervical epithelial tissues express a high level of FREM1 mRNA [15], and TILRR is an isoform of FREM1 [60] and a major modulator of many inflammatory responsive genes in the NF-κB pathway [16], the expression of FREM1/TILRR in genital epithelial tissues may augment the inflammatory responses via NF-κB activation and the production of inflammatory mediators. We, therefore, propose a model depicting the potential roles of FREM1/TILRR in vaginal HIV-1 acquisition through mediating inflammation (Figure 4A–D) based on our in vitro studies [16,69], a genetic study [15] and a number of related publications [24,25,47,58,70].

In this model, we propose that the physiological level of FREM1/TILRR expression in healthy female genital tissues maintains the epithelial integrity, which prevents the invasion of pathogens, including HIV-1 into the genital tissues [73] (Figure 4B). In HIV-1 susceptible female genital tissues, on the other hand, the high-level expression of FREM1/TILRR increases the expression of different inflammation responsive genes through activation of NF-κB signaling pathway, and production of inflammatory cytokines/chemokines by epithelial cells (Figure 4C). The increased production of inflammatory cytokines/chemokines attracts the infiltration of immune cells, including CD4+T cells and monocytes/macrophages, the HIV-1 target cells, from the periphery to the genital tissue. The increased HIV-1 target cells will increase the chance for the HIV-1 to establish infection. Additionally, cytokines/chemokines secreted by the infiltrated immune cells may further activate and attract more HIV-1 target cells and resulted in cycles of inflammation and breakdown of the epithelial barrier (Figure 4C). Thus, the proposed model showed that the elevated expression of FREM1/TILRR promotes the increased production of inflammatory mediators, increased infiltration of HIV-1 target cells, and profound inflammatory responses resulting in the breakdown of epithelial integrity and HIV-1 susceptibility (Figure 4D).

## 5. Conclusions and Future Direction

FREM1 and TILRR in cervical epithelial tissues may play a potential role in vaginal HIV-1 infection. FREM1 isoform TILRR augments the mRNA expression of many inflammatory genes, production of inflammatory mediators, and migration of immune cells (HIV-1 target cells). Our proposed model will open new avenues to further study the role of FREM1 and TILRR in genital inflammation and vaginal HIV-1 acquisition in women. Future studies using in vivo model(s) may identify novel interventions to control HIV-1 acquisition, transmission, and disease progression.

## Supplementary Materials

The following are available online at https://www.mdpi.com/article/10.3390/ijms22157825/s1.
